# Assessment of Breastfeeding, Weaning, and Complementary Feeding Practices Among Women Attending a Tertiary Care Teaching Hospital in South India

**DOI:** 10.7759/cureus.28791

**Published:** 2022-09-05

**Authors:** Ayesha Jabeen, Amtul Rahman Amberina, Vemula Gayathri, Mummareddi Dinesh Eshwar, Saikrishna Dodda, Gulam Saidunnisa Begum, Sabitha Vadakedath, Venkataramana Kandi

**Affiliations:** 1 Biochemistry, Mahavir Institute of Medical Sciences, Vikarabad, IND; 2 Medicine, Navodaya Medical College, Raichur, IND; 3 Biochemistry, College of Medicine and Health Sciences, Sohar, OMN; 4 Biochemistry, Prathima Institute of Medical Sciences, Karimnagar, IND; 5 Clinical Microbiology, Prathima Institute of Medical Sciences, Karimnagar, IND

**Keywords:** health, infant, children, complementary food, weaning, breastfeeding

## Abstract

Introduction

Infants depend exclusively on mothers’ milk for nutrition in their early months of life. However, some infants are given cow/buffalo milk during insufficiency. After six months, the children are supplemented with complementary food, including solid food, to meet the nutritional requirements of a growing infant, a process known as weaning. Therefore, it is important for mothers to have a clear understanding of the nature of food, and the nutritional requirements of the children. This study aimed to understand the knowledge, awareness, and practice of breastfeeding, weaning, and complementary feeding among women.

Methods

A cross-sectional questionnaire-based observational study was carried out between May and June 2021. The study included 150 women attending the outpatient department of pediatrics attached to Mahavir Institute of Medical Sciences (MIMS), Vikarabad, Telangana, India. After taking the informed consent, the researcher filled out a pre-validated questionnaire based on the subjects' responses. The data regarding socio-demographic details and information regarding knowledge, timing, practices of weaning, knowledge of nutritional requirements, and child feeding practices were collected as a pre-test. An educational briefing of the essentials of child feeding practices, weaning, and dietary requirements was undertaken using chalk and board and audio-visual aids, among others. In the post-test, the knowledge of mothers regarding weaning practices, the importance of weaning, and understanding of the nutritional requirements and their inclusion in a child’s diet was assessed. The quantitative data were represented as percentages. The Chi-square test was applied to find out the statistical significance of the results obtained from the study.

Results

Of the 150 women who participated in the study, the majority belonged to the age group of 18-23 years (66.7%). Most women were illiterate (34%) and only 12% were graduates. More than 70% of the participants belonged to the middle and lower middle class. The majority of participants (96.7%) had carried out exclusive breastfeeding for the first six months, and 63.35% of mothers had initiated weaning their child between the age of 6-12 months. Only 37.4% of mothers started breastfeeding within hours after the delivery. A combination of all foods (36%), rice/wheat (29%), processed food (18.8%), fruits and vegetables (9.2%), and pulses (6.7%) were chosen for weaning. Many felt that eggs and meat supplementation can be done between 12-18 months.

Conclusion

The knowledge of breastfeeding and best practices of weaning and complementary feeding habits significantly affects the child’s growth and overall health. The women in this study had a moderate level of understanding regarding the aspects of breastfeeding, weaning practices, and complementary dietary requirements during weaning.

## Introduction

Breastfeeding for the first six months of life, time to weaning, and the quality of complementary food during weaning significantly affect the lifelong eating behaviors and health status of children. However, people belonging to low socioeconomic classes, illiterates, and those who reside in rural areas with minimal access to healthcare do not have adequate knowledge of weaning practices and the nutritional requirements of children.

Breastfeeding the children exclusively during the first six months of birth confers the baby with a passive immunity that is essential in the absence of a well-developed immune system. Moreover, the children are supplemented with complementary food after six months to meet the increasing nutritional requirements necessary for growth and development. This process is defined as weaning, and mothers who carry out weaning must be well informed about the timing and types of complementary food along with other aspects of nutrition [1, 2].

Improper nutrition could predispose children to irreversible cognitive damage and affects their physical and psychological health. Considering the importance of complementary food and because there is no specific guideline about the composition and quality of such food, it is essential for mothers to improve their knowledge. Moreover, the low nutritional value of complementary food predisposes children to stunted growth, low immune responses, and cardiovascular diseases [3]. Recent research has pointed toward the positive effects of nutrition-rich complementary food in the development of a beneficial gut microbiome and a healthier respiratory system [4, 5]. The withdrawal of breastfeeding, time to weaning, and the nature of complementary feeding differ with the social, cultural, economic, and geographical patterns globally [6, 7].

This study was carried out to explore the knowledge, awareness, and practice of breastfeeding, weaning, and complementary feeding habits of women in the rural parts of Telangana, South India.

## Materials and methods

A cross-sectional questionnaire-based observational study was carried out among mothers attending the outpatient section of the department of pediatrics attached to Mahavir Institute of Medical Sciences (MIMS), Vikarabad, Telangana, India. The study was carried out for a period of two months (May-June, 2021). A total of 150 women were enrolled in the study after taking informed consent, and the study was approved by the Institutional Ethics Committee of MIMS (MIMS/IEC/2021/March). The women who had children less than five years of age were included in the study. All women who had children diagnosed with inborn metabolic disorders, diabetes, heart disease, and infants who were on special feeds or diets were excluded from the study.

A pre-validated questionnaire was used to collect the data from all the study participants (see Appendices). The data included were socio-demographic details and information regarding knowledge, timing, and practices of breastfeeding and weaning, and the nutritional importance of complementary food. This was followed by a 30-minute briefing on the standard and recommended practices of breastfeeding, weaning, and complementary foods wherein chalk and board, audio-visual aids, and internet resources (YouTube videos, among others) were used. Later, a post-test questionnaire was used to evaluate the knowledge of breastfeeding, weaning, and complementary feeding practices of women included in the study.

Statistical analysis

The data obtained were entered into Microsoft Office 2019 Excel sheet (Microsoft® Corp., Redmond, WA), and statistical inferences were drawn using SPSS software version 24 (IBM Corp., Armonk, NY). The quantitative data were represented as percentages. The Chi-square test was applied to find out the statistical significance of the results obtained from the pre-test and post-test. The significance threshold of the p-value was set at <0.05.

## Results

Of the total 150 participants included in the study, the majority (66.3%) were women in the age group of 18-23 years followed by women in the 24-29 years age group (26.7%). The bulk of the women included in the study was illiterate (34%), followed by those who were primary skilled (24.7%), moderately skilled (12.7%) and only a very few were graduates (12%). The majority of the women belonged to the middle class (45.3%), followed by the lower middle class (22.6%), and lower class (18.7%). The demographic details of the study participants are delineated in Table 1.

**Table 1:** Demographic data of the study participants

Variable		n (%) (Total=150)
Age (years)	<18	03 (2%)
	18-23	100 (66.7%)
	24-29	40 (26.7%)
	≥30	07 (4.6%)
Mother’s Education/skill levels	Pre-university	08 (5.3%)
	Graduate	18 (12%)
	Diploma	05 (3.3%)
	Highly skilled	12 (8%)
	Moderately skilled	19 (12.7%)
	Low skilled	37 (24.7%)
	Illiterate	51 (34%)
Socioeconomic status	Upper class	07 (4.7%)
	Upper middle class	28 (18.7%)
	Middle class	68 (45.3%)
	Lower middle class	34 (22.6%)
	Lower class	13 (18.7%)

Many women (37.4%) initiated breastfeeding immediately after the delivery. However, a significant number of women (31.3%) initiated breastfeeding within 24 hours, and an equal number of women initiated breastfeeding after 24 hours of delivery. The majority of participants (96.7%) had carried out exclusive breastfeeding for the first six months, and 63.35% of mothers had initiated weaning their child between the age of 6-12 months.

Women in this study looked for the baby to be able to sit up (62%) before they start the weaning. Other signs identified by the women before weaning included signs of the baby being able to hold their hands up (27.3%) and roll over (10.7%) before initiating the weaning process. Nearly 54% of women gave a combination of all foods including cereals, pulses, and fruits. However, a few fed their children with rice/wheat (29.3%), and processed foods (18.8%).

The details of the breastfeeding and weaning practices among the study participants are given in Table 2.

**Table 2:** Knowledge and practice of breastfeeding and weaning practices among study participants *Statistically significant

Variable	Choice	Pre-test n (%)	Post-test n (%)	p-value
Time of initiation of breastfeeding	Immediately after delivery	56 (37.4)	137 (90)	<0.0001*
	Within 24 hours	47 (31.3)	10 (6.6)	
	After 24 hours	47 (31.3)	5 (3.3)	
Exclusive breastfeeding for 6 months	Yes	145 (96.7)	150 (100)	<0.0001*
	No	05 (3.3)	0 (0)	
At what age would you start weaning your child	<6 months	10 (6.7)	0 (0)	<0.0001*
	6-12 months	95 (63.3)	150 (100)	
	12-18 months	33 (22)	0 (0)	
	>18 months	12 (8)	0 (0)	
The signs you look for before starting weaning	Hold their hands up	41 (27.3)	128 (85.3)	<0.0001*
	Sit up	93 (62)	22 (14.7)	
	Roll over	16 (10.7)	0 (0)	
Preferred food while weaning	Rice/wheat	44 (29.3)	20 (13.3)	<0.0001*
	Pulses	10 (6.7)	5 (3.4)	
	Fruits/vegetables	14 (9.2)	10 (6.7)	
	Combined diet	54 (36)	113 (75.3)	
	Processed/canned food	28 (18.8)	2 (1.3)	
Do you minimize breastfeeding during weaning	Yes	35 (23.3%)	12 (8%)	<0.0001*
	No	115 (76.7%)	138 (92%)	

Most women considered semisolid as a preferred consistency (68.7%), followed by liquid food (25.3%) and a minority of them preferred a solid consistency (6%). Regarding the quantity of food, 44% of women considered a half cup sufficient. However, 40% of women fed their child with one-quarter of the cup and 16% fed a cup full of food. The number of complementary feeds per day for most of the children was three times (54.7%) followed by less than two times feeding per day (34%) and more than three times feeding per day (11.3%). The details of the complementary feeding habits among the study participants are delineated in Table 3.

**Table 3:** Knowledge and practice of complementary food during weaning *Statistically significant

Variable	Choice	Pre-test n (%)	Post-test n (%)	p-value
Consistency of complementary feeds	Liquid	38 (25.3)	03 (2)	<0.0001*
	Semisolid	103 (68.7)	10 (6.6)	
	Solid	147 (98)	09 (6)	
The amount of weaning food is given per serving	1/4^th^ cup	60 (40)	01 (0.7)	<0.0001*
	Half cup	66 (44)	90 (66)	
	One cup	24 (16)	59 (33.3)	
Number of complimentary feeds per day	<2 times	51 (34)	0 (0)	<0.0001*
	3 times	82 (54.7)	0 (0)	
	>3 times	17 (11.3)	0 (0)	
Are pureed fruits and vegetables good for weaning	Yes	148 (98.7)	149 (99.3)	<0.0001*
	No	01 (0.7)	2 (1.3)	
When did you start feeding the egg?	<6 months	01 (0.7)	0 (0)	<0.0001*
	6-12 months	35 (23.3)	138 (92)	
	12-18 months	84 (56)	12 (8)	
	>18 months	30 (20)	0 (0)	
When did you start feeding meat?	<6 months	0 (0)	0 (0)	<0.0001*
	6-12 months	18 (12)	137 (91.3)	
	12-18 months	60 (40)	13 (8.7)	
	>18 months	72 (48)	0 (0)	

## Discussion

According to the World Health Organization (WHO), infant mortality rates are high in the African (43%), Central, and South-East Asian (36%) countries. It has also been observed that a majority of neonatal deaths occur within 28 days and mortality was associated with a lack of quality care in the early days after birth. However, significant numbers of children also die due to infections, malnutrition, and other factors. Moreover, India stands top in the list of countries that recorded the highest number of newborn deaths with 490,000 deaths in the year 2020. The WHO, therefore, has recommended that mothers, families, and other caregivers should be empowered with quality newborn care [8].

The global infant mortality data suggests that more than half of deaths among children under five years of age were from five countries that included India, Pakistan, Nigeria, the Democratic Republic of the Congo, and Ethiopia. Moreover, India and Nigeria combined accounted for one-third of mortality as estimated by the United Nations Inter-agency Group for Child Mortality Estimation (UN IGME) [9].

Because breastfeeding immediately after birth ensures passive immunity, the baby is protected against most infections. However, during the process of weaning, the children are supplemented with complementary food. Therefore, it is important that the children are provided nutrition-rich food that contributes to the development of a robust immune system.

The study results have demonstrated that the women from this geographical region have a moderate level of knowledge of breastfeeding, weaning, and complementary feeding practices. The levels of awareness among this population have significantly improved with the briefing of standard and recommended breastfeeding, weaning, and complementary food practices. Complementary feeding is also known alternatively as the baby-led weaning process (BLW). It was noted that BLW is more predominantly practiced among educated mothers. However, due to fears of choking, mothers have been extremely cautious and selective in terms of the consistency and type of complementary food that may directly affect the intake of essential minerals and vitamins [10].

A satisfactory level of breastfeeding and infant feeding practices was noted among the tribal women belonging to the Eastern parts of India. More than 78% of women were noted to practice exclusive breastfeeding and 82% of them initiated complementary feeding after six months. The exclusive breastfeeding practice in our study was significantly higher (96.7%) owing to the inclusion of non-tribal women residing in rural areas [11].

In a study from Malawi, 30.8% of children were noted to suffer from stunted growth. This was associated with lower rates of exclusive breastfeeding practice as evidenced by the Malawi Demographic Health Survey (2015-2016) study that assessed 2294 children aged between 0-23 months. This study also noted that women in urban areas are less likely to breastfeed infants immediately after birth. However, they follow better complementary feeding practices [12].

The knowledge of weaning was noted to be unsatisfactory among the women from Saudi Arabia. Moreover, women were not adequately educated about the symptoms of weaning among infants. Despite adequate educational qualifications, the infants were underfed. This was attributed to the fact that women preferred to follow local customs instead of seeking doctors' advice [13].

Poor knowledge of weaning (6.2%) and complementary feeding practices was observed in a study that was reported from Nigeria. It was noted that the age of the mother and the family settings significantly influenced the knowledge and practice of weaning and complementary feeding [14]. In a study from Turkey, it was noted that only 44% of women followed exclusive breastfeeding despite having the knowledge that six-month exclusive breastfeeding is essential for children. More than 90% of mothers preferred formula food as a source of complementary food [15].

After exclusive breastfeeding, the timing of the introduction of complementary food was noted to influence the growth velocity of the children as observed from the results of a study from the United States of America (USA) [16]. Complementary feeding practices among South Asian people revealed that most mothers initiated complementary feeding between 6 and 12 months. This large-scale study revealed suboptimal feeding practices, with the diversity of complementary food lower than 50%. Moreover, complementary feeding practices were influenced by the cultural backgrounds, poor knowledge, and educational qualifications of the mothers [17].

The quality of the complementary food is extremely important since contamination of food predisposes children to gastrointestinal parasitic infections as noted from the results of a previous study [18]. Exclusive breastfeeding was replaced by formula milk, and the perception of weaning age, and the nutritional value of the complementary food were associated with the levels of the mother’s knowledge, especially in semi-rural settings and among working women [19]. Mothers of children aged under five years are required to be well informed about the significance of exclusive breastfeeding, and the nutritional value of the complementary food. Best infant and young child feeding (IYCF) practices are essential to minimize the morbidity and mortality rates among neonates and children as suggested by the WHO [20].

Study limitations

The major drawback of this study was the inclusion of fewer sample numbers. Moreover, the causes and potential barriers to moderate-level knowledge were not investigated. Also, the children were neither included, nor evaluated for medical consequences as a result of improper breastfeeding, weaning, and complementary feeding practices.

## Conclusions

The majority of mothers followed exclusive breastfeeding and introduced complementary foods to their infant’s diet at the recommended age of six months. However, the quality of complementary food, composition, quantity, and frequency was found to be inadequate. Moreover, the majority of the uneducated mothers withheld complementary food from their babies which was attributed to their lack of education and knowledge. The awareness regarding the feeding practices was poor, which significantly increased after the educational intervention as reflected in the post-test results. The delayed introduction of semisolid food could, in the future, contribute to the development of protein-energy malnutrition and result in a high incidence of infant mortality. Implementation of awareness campaigns, surveys, and other educational interventions are required to increase the knowledge regarding best practices in breastfeeding, weaning, and complementary feeding.

## Appendices

PRE-TEST

DEMOGRAPHIC DETAILS:

I.        Name of the child           :

II.     Age of the child              :

III.   Is the child vaccinated appropriately to Age? Yes / NO

IV.  Age of the mother          :

V.     Address                           :

VI.  Socioeconomic Status: 1) Upper class (≥36000$ per annum) 2) Upper middle class (15,000-35000$ per annum) 3) middle class (5000-14000$ per annum) 4)  lower middle class (1000-5000$ per annum) 5) Lower (<1000$ per annum)

VII.       Mother’s Education: 1) Pre-university degree 2) Graduate/degree 3) Diploma 4) High skilled

5) Middle skilled 6) Primary skilled 7) Illiterate

QUESTIONNAIRE:

1) Time of initiation of Breast Feeding

1.     Immediate after labor

2.     Within 24 hours

3.     After 24 hours 2) Colostrum Feeding? Yes / No

3) Exclusive Breastfeeding for 6 months? Yes / No 4) At what age would you start weaning your child?

1.     Less than 6 months

2.     6 to 12 months

3.     12 to 18 months

4.     Greater than 18 months

5) What did you give your baby to start weaning

1.     Rice/wheat

2.     Pulses

3.     Fruits and vegetables

4.     Combination of all foods

5.    Processed food (cereals, biscuits, noodles)

6) What is the consistency of your complementary feeds?

1.     Watery

2.     Semisolid

3.     Solid

7) When do you start giving egg?

1.     Less than 6 months

2.     6 to 12 months

3.     12 to 18 months

4.     Greater than 18 months

8) When do you start giving Meat/Non-Veg

1.     Less than 6 months

2.     6 to 12 months

3.     12 to 18 months

4.     Greater than 18 months

9)  How much amount of weaning food is given per serving after initiation of weaning?

1.     One-fourth small cup

2.     Half small cup

3.     Small cup

10) No. of complementary feeds per day?

1.     <2 Times

2.     3 Times

3.     >3 Times

11) Do you think purified fruits and vegetables are good for weaning babies? Yes / No

12) Do you reduce milk feeds once you start weaning? Yes / No

13) What should your baby be able to do before you start weaning

1.     Hold their head up

2.     Sit-up

3.     Rollover

POST-TEST

QUESTIONNAIRE:

1) Time of initiation of Breast Feeding

1.     Immediate after labour

2.     Within 24 hrs

3.         After 24 hrs

2) Colostrum Feeding? Yes / No

3)   Exclusive Breastfeeding for 6 months? Yes / No 4) At what age should a mother start weaning a child?

1.     Less than 6 months

2.     6 to 12 months

3.     12 to 18 months

4.     Greater than 18 months

5) What should be given for weaning a baby

1.     rice/wheat

2.     pulses

3.     fruits and vegetables

4.     combination of all foods

5.     processed food (cereals, biscuits, noodles) 6) What is the consistency of complementary feeds?

1.     watery

2.     semisolid

3.     solid

7) When do you start giving egg?

1.     Less than 6 months

2.     6 to 12 months

3.     12 to 18 months

4.     Greater than 18 months

8) When do you start giving Meat/NonVeg

1.     Less than 6 months

2.     6 to 12 months

3.     12 to 18 months

4.     Greater than 18 months

9) How much amount of weaning food is given per serving after initiation of weaning?

1.     One-fourth small cup

2.     Half small cup

3.     Small cup

10) No. of complementary feeds per day?

1.     <2 Times

2.     3 Times

3.     >3 Times

11)  Are pureed fruits and vegetables good for weaning babies? Yes / No

12)  Do you reduce milk feeds once you start weaning? Yes / No 13) What should your baby be able to do before you start weaning

1.     Hold their head up

2.     Sit-up

3.     Rollover

Informed Consent Form

Title of Project:

I, Mrs. /Miss/Mr.                                                give my consent to be part of this study.

I have read and understood the information provided or it has been read and explained to me.

I have had the opportunity to ask questions about the research and all the questions that I have asked have been answered to my satisfaction. I consent to be a participant in this research work.

I also understand that participation in this study is completely voluntary and I have been informed that I can withdraw from the study anytime if I decided not to participate.

Signature of Participant:                                                                                                              Age:                                Date:                                  Time:                                 

If participant is unable to give consent

I have read the provided information or it has been read and explained to me. I have had the opportunity to ask questions about the research and all the questions that I have asked have been answered to my satisfaction. I give my permission for participant to take part in this research work.

Name of the Legal Representative:                                                                                             Date:                              Time:                                       Signature:                                                          Statement by researcher/person taking consent

I have correctly read out the information sheet to the participant, and to the best of my ability made sure that the participant understands the information included. I confirm that the participant was given an opportunity to ask questions about the study, and all questions asked by the participant have been answered correctly and to the best of my ability. I confirm that the individual has not been pressurized, into giving consent, and the consent has been given freely and voluntarily.

Name of the researcher: Date:                Time:   Signature:                    
